# Not Just Another Broken Heart: A Case Report of Takotsubo Cardiomyopathy Causing Syncope

**DOI:** 10.5811/cpcem.47179

**Published:** 2025-10-22

**Authors:** Aileen Virella, Stephanie Jose, Joseph Mirro, Allison Cohen, Nicholas Bielawa, Mathew Nelson

**Affiliations:** North Shore University Hospital, Department of Emergency Medicine, Manhasset, New York

**Keywords:** syncope, takotsubo syndrome, left ventricular outflow tract obstruction, point-of-care ultrasound, case report

## Abstract

**Introduction:**

Patients with symptoms suggestive of acute coronary syndromes account for up to 10% of emergency department (ED) visits, and of those visits 2% are diagnosed with takotsubo syndrome. Takotsubo syndrome associated with left ventricular outflow tract (LVOT) obstruction is an important but uncommon cause of chest pain and syncope in patients presenting with ST-segment elevations. Although rare, this variant is associated with worse clinical outcomes. Early recognition of LVOT obstruction in these patients is important to help guide proper management.

**Case Report:**

We report a case of a 66-year-old female presenting to the ED after a syncopal episode with ST-segment elevations on the electrocardiogram. Point-of-care ultrasound revealed apical hypokinesis, thickened basal septum with LVOT obstruction and systolic anterior motion.

**Conclusion:**

Point-of-care ultrasound can help quickly diagnose takotsubo cardiomyopathy and its complications, providing guidance to accurate management.

## INTRODUCTION

Syncope is a common cause of emergency department (ED) visits. While the underlying etiology is often benign, approximately 10% of these patients have a more serious underlying condition requiring further work-up and diagnostic evaluation.^1^ Potentially life-threatening cardiac causes of syncope, including arrhythmias, structural abnormalities, and ischemia, are often diagnosed in the ED.^2^ Among patients presenting with syncope, those with abnormal electrocardiograms (ECG) and symptoms suggestive of acute coronary syndrome, may actually be caused by takotsubo syndrome with left ventricular outflow tract (LVOT) obstruction. Takotsubo syndrome, also known as broken heart syndrome, is a form of cardiomyopathy that can resemble a myocardial infarction, presenting with similar clinical symptoms, ECG changes, and echocardiogram findings.^3^ Although a rare presentation, patients presenting with syncope due to dynamic LVOT obstruction require unique management considerations. We discuss a case that highlights the critical role of point-of-care ultrasound (POCUS), specifically echocardiogram, in the early diagnosis of takotsubo syndrome in the ED to further guide management.

## CASE REPORT

**Image 1:**
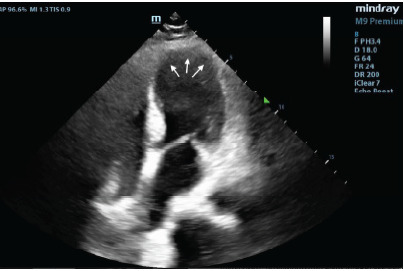
Echocardiogram of the apical four-chamber view using a low-frequency phased array probe showing left ventricular apical thinning with ballooning of the wall (arrows).

**Image 2:**
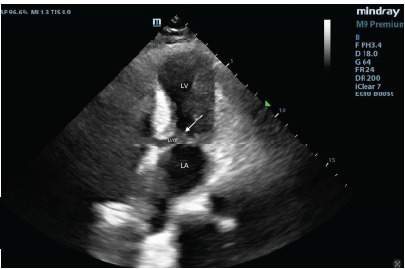
Echocardiogram of the apical five-chamber view, showing the left ventricle (LV), left atrium (LA), left ventricular outflow tract (LVOT), and the anterior mitral valve leaflet (arrow) obstructing the LVOT during systole.

A 66-year-old female with a medical history of hypertension and hyperlipidemia presented to the ED following a syncopal episode during exercise. The patient was participating in a Zumba class when she experienced lightheadedness, followed by a syncopal event, resulting in trauma to the right side of her face and right upper arm. Emergency medical services (EMS) reported an abnormal ECG with ST-segment changes. She was also noted by EMS to be hypotensive and received a small volume of intravenous fluids. The patient denied chest pain or shortness of breath but reported experiencing intermittent lightheadedness over the prior one to two weeks while walking, which resolved with rest. Upon arrival, the patient was awake, alert, and responsive to commands, with vital signs within normal limits. The physical examination was notable for a grade 3 harsh systolic murmur heard best at the left sternal border, while the remainder of her exam was unremarkable. A POCUS was then performed to further evaluate the myocardial function. The ultrasound revealed anterior and apical hypokinesis with ballooning of the apex and thickened basal septum with LVOT obstruction (Image 1) and systolic anterior motion (SAM) (Image 2).

**Image 3:**
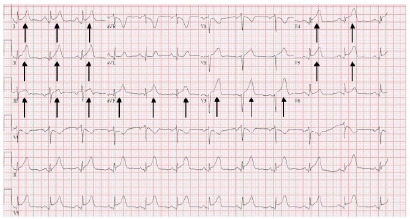
Electrocardiogram revealing diffuse ST-segment elevations (arrows).

Laboratory findings showed a significant elevation in troponin T from 22 to 222 nanograms per liter (ng/L) (reference range: 0–51 ng/L) within one hour and a creatine kinase MB of 8.2 ng/mL (0.0–3.8.ng/mL). Initial ECG demonstrated a normal sinus rhythm at a rate of 68 beats per minute with diffuse ST elevations and PR depressions (Image 3). Cardiology was consulted out of concern for an ST-elevation myocardial infarction.

After reviewing the POCUS, the decision was made to take the patient to the catheterization lab, due to anterior apical wall motion abnormalities after computed tomography of the head was performed to rule out traumatic injury. Findings from the catheterization revealed no significant coronary artery disease. She was subsequently admitted to the cardiac intensive care unit, where she required a phenylephrine drip for persistent hypotension secondary to takotsubo cardiomyopathy with LVOT obstruction and SAM of the mitral valve. The patient was eventually weaned off vasopressors with fluid therapy and discharged home on a beta blocker after a four-day hospital stay.

*CPC-EM Capsule*

What do we already know about this clinical entity?

*Takotsubo syndrome is a reversible cardiomyopathy but can sometimes cause left ventricular outflow tract (LVOT) obstruction, a complication associated with worse outcomes*.

What makes this presentation of disease reportable?

*Point-of-care ultrasound (POCUS) helped in diagnosing takotsubo syndrome with LVOT obstruction in a patient with syncope and electrocardiogram changes*.

What is the major learning point?

*Takotsubo syndrome can lead to cardiogenic shock and arrythmia. POCUS is key to making the diagnosis and evaluating a patient presenting with syncope*.

How might this improve emergency medicine practice?

*POCUS is highly useful in diagnosing the cause of life-threatening syncope*.

## DISCUSSION

Takotsubo syndrome is a well-recognized acute, reversible myocardial injury characterized by transient regional cardiac dysfunction, believed to result from either increased catecholamine-induced myocyte toxicity and ischemia, or in patients with hypertrophic cardiomyopathy due to latent LVOT obstruction.^5^ Takotsubo syndrome exhibits distinct features that differentiate it from other acute cardiac emergencies. It is defined by non-obstructed coronary arteries and a distinct anteroseptal-apical dyskinetic ballooning of the left ventricle, accompanied by hyperkinetic basal segments. This characteristic shape resembles an inverted vase, like the traditional pots used by Japanese fishermen to trap octopuses, which inspired the syndrome’s name.^6^ Initially, it was considered a rare event; however, with increasing physician awareness the incidence of takotsubo syndrome is estimated to affect approximately 2% of patients being evaluated for acute coronary syndrome.^6^

Risk factors for the syndrome include diabetes, post-menopausal status, cannabis use disorder, and recent asthma exacerbation.^7^ Although often considered a benign and often self-limiting syndrome, takotsubo syndrome can frequently lead to complications such as arrythmias, cardiogenic shock, heart failure, and valvulopathies including mitral regurgitation from SAM of the mitral leaflet, making early identification important.^2^,^5^ Patients with LVOT obstruction and SAM represent the most severe form of takotsubo syndrome and often present the greatest challenges in management and treatment.^7^

Systolic anterior motion refers to the displacement of the distal anterior mitral leaflet toward the interventricular septum during ventricular systole, causing obstruction. Due to the characteristic pattern of regional wall motion abnormalities in takotsubo syndrome, as previously mentioned, the anterior mitral leaflet is subjected to the Venturi effect. This phenomenon occurs when drag forces pull the leaflet forward, leading to contact with the septum, subaortic obstruction, and posteriorly directed mitral regurgitation into the left atrium.^8^

Point-of-care echocardiography plays a crucial role in diagnosing SAM, with M-mode providing direct visualization of the anterior displacement of the mitral valve during systole.^10^ The primary approach to managing SAM involves medical therapy, particularly the use of negative inotropic agents such as non-vasodilating beta blockers to alleviate outflow obstruction.^9^ Additional strategies include volume resuscitation to improve preload and ventricular filling and peripheral alpha-adrenergic stimulation (eg, phenylephrine).^10^ Inotropes should be avoided or discontinued, as they can exacerbate dynamic LVOT obstruction.^9^

Clinically, the initial presentation and ECG changes in these patients mimic a myocardial infarction, leading to the initiation of heparin and, in some cases, inotropes to manage associated low blood pressure. Additionally, patients presenting with syncope caused by cardiac arrythmia, ischemia, or structural abnormalities face a higher risk of adverse outcomes, making earlier identification critical for improving care.^11^ Although patients with takotsubo syndrome are less likely to present with syncope, it is estimated that 7–25% of patients of these patients display LVOT obstruction.^4^ Therefore, recognizing the distinctive features of takotsubo syndrome via point-of-care echocardiogram in the ED is crucial for accurate diagnosis and effective management, allowing for prevention of hemodynamic compromise and cardiogenic shock. Early identification also helps clinicians avoid treatments commonly used for acute coronary syndrome such as nitrates, afterload-reducing agents, and inotropes, which can worsen LVOT obstruction.^12^,^13^

## CONCLUSION

Rapid identification of life-threatening causes of syncope, including cardiac arrythmias, ischemia, or cardiac structural abnormalities, is crucial for emergency physicians. One known cause of syncope is left ventricular outflow tract obstruction, which can result from takotsubo syndrome or hypertrophic cardiomyopathy. Although uncommon, LVOT obstruction is a potentially serious complication of takotsubo syndrome, often presenting with abnormal ECG findings and hemodynamic instability. Emergency physicians should consider this complication when evaluating patients presenting after syncope with an abnormal ECG and a concerning history, especially when point-of-care ultrasound reveals characteristics such as apical ballooning, hypokinesis, and evidence of LVOT obstruction. This case further highlights the value of POCUS in expanding differential diagnoses and detecting abnormalities, ultimately leading to accurate patient management and treatment, as well as improved patient outcomes.

## References

[1] Wakai A, Sinert R, Zehtabchi S (2025). Risk-stratification tools for emergency department patient with syncope: a systematic review and meta-analysis of direct evidence for SAEM GRACE. Acad Emerg Med.

[2] Khoo C, Chakrabarti S, Arbour L (2013). Recognizing life-threatening causes of syncope. Cardiol Clin.

[3] Goodacre S, Cross E, Arnold J (2005). The health care burden of acute chest pain. Heart.

[4] Lee L, Khawcharoenporn T, Chokrungvaranon N (2009). Takotsubo cardiomyopathy associated with syncope. Heart Lung.

[5] Citro R, Bellino M, Merli E (2023). Obstructive hypertrophic cardiomyopathy and takotsubo syndrome: how to deal with left ventricular ballooning. J Am Heart Assoc.

[6] Singh T, Khan H, Gamble D (2022). Takotsubo syndrome: pathophysiology, emerging concepts, and clinical implications. Circulation.

[7] Medina de Chazal H, Del Buono MG, Keyser-Marcus L (2018). Stress cardiomyopathy diagnosis and treatment: *JACC* state-of-the-art review. J Am Coll Cardiol.

[8] Wigle D, Rakowski H, Kimball P (1995). Hypertrophic cardiomyopathy. Clinical spectrum and treatment. Circulation.

[9] Veselka J, Anavekar NS, Charron P (2017). Hypertrophic obstructive cardiomyopathy. Lancet.

[10] Loulmet F, Yaffee W, Ursomanno A (2014). Systolic anterior motion of the mitral valve: a 30-year perspective. J Thorac Cardiovasc Surgy.

[11] Puppala VK, Dickinson O, Benditt DG (2014). Syncope: classification and risk stratification. J Cardiol.

[12] Haley JH, Sinak LJ, Tajik AJ (1999). Dynamic left ventricular outflow tract obstruction in acute coronary syndromes: an important cause of new systolic murmur and cardiogenic shock. Mayo Clin Proc.

[13] Luria D, Klutstein MW, Rosenmann D (1999). Prevalence and significance of left ventricular outflow gradient during dobutamine echocardiography. Eur Heart J.

